# Reconfiguration of human evolving large-scale epileptic brain networks prior to seizures: an evaluation with node centralities

**DOI:** 10.1038/s41598-020-78899-7

**Published:** 2020-12-14

**Authors:** Rieke Fruengel, Timo Bröhl, Thorsten Rings, Klaus Lehnertz

**Affiliations:** 1 Department of Epileptology, University Hospital Bonn, Venusberg Campus 1, 53127 Bonn, Germany; 2 Helmholtz-Institute for Radiation and Nuclear Physics, University of Bonn, Nussallee 14-16, 53115 Bonn, Germany; 3 Interdisciplinary Center for Complex Systems, University of Bonn, Brühler Straße 7, 53175 Bonn, Germany

**Keywords:** Neurology, Physics

## Abstract

Previous research has indicated that temporal changes of centrality of specific nodes in human evolving large-scale epileptic brain networks carry information predictive of impending seizures. Centrality is a fundamental network-theoretical concept that allows one to assess the role a node plays in a network. This concept allows for various interpretations, which is reflected in a number of centrality indices. Here we aim to achieve a more general understanding of local and global network reconfigurations during the pre-seizure period as indicated by changes of different node centrality indices. To this end, we investigate—in a time-resolved manner—evolving large-scale epileptic brain networks that we derived from multi-day, multi-electrode intracranial electroencephalograpic recordings from a large but inhomogeneous group of subjects with pharmacoresistant epilepsies with different anatomical origins. We estimate multiple centrality indices to assess the various roles the nodes play while the networks transit from the seizure-free to the pre-seizure period. Our findings allow us to formulate several major scenarios for the reconfiguration of an evolving epileptic brain network prior to seizures, which indicate that there is likely not a single network mechanism underlying seizure generation. Rather, local and global aspects of the pre-seizure network reconfiguration affect virtually all network constituents, from the various brain regions to the functional connections between them.

## Introduction

Epilepsy is one of the most common neurological diseases globally, affecting an estimated 50 million people worldwide, and accounting for 0.5% of the global burden of disease^1^. Even in countries where adequate diagnosis and treatment are available, around 30% of epilepsies are pharmacoresistant, failing to respond to conventional medical therapy^2^. In these cases, subjects with epilepsy may be candidates for surgical intervention, which allows around 70% of these subjects to remain seizure-free for at least 1 year after surgery^3^. Among several other aspects, this failure to achieve long-lasting freedom from seizures, even after removal of the pre-surgically identified seizure onset zone (SOZ), suggests an alternative interpretation of seizure generation (ictogenesis) in epilepsy.

In recent years, epilepsy has been investigated as a network disease^4–7^. In a large-scale evolving epileptic brain network, sampled brain regions represent nodes, whereas the time-varying functional interactions between them (regardless of their anatomical connectedness) constitute the time-dependent edges of the network^8^. This results in a sequence of networks that evolve in time. When considering the SOZ as a node (or a small group of nodes) in the evolving epileptic brain network, previous studies reported the SOZ to play only a minor role in seizure dynamics^9,10^, in contrast to earlier observations^11–13^. A more recent study^14^ of evolving epileptic brain networks has identified nodes, whose time-dependent changes in node centrality carry predictive information about an impending seizure. More importantly, these predictive nodes were exclusively associated with brain regions far away from the SOZ, in accordance with a number of previous findings achieved with different analysis concepts^15^. This study indicated a reconfiguration of various network properties of evolving epileptic brain networks during the pre-seizure period, which is not confined to nodes related to the SOZ but extends to the whole network. A more detailed characterisation of node centrality can aid in understanding this reconfiguration and subsequently can help to shed more light on how seizures arise from epileptic brain networks. Indeed, a large number of different centrality indices have been developed to characterise the various roles the constituents play in the network^16–18^. Here, we consider four of the most widely used centrality indices^17^, two different interaction-strength-based centrality indices (strength centrality $\mathcal {C}^\text{S}$ and eigenvector centrality $\mathcal {C}^\text{E}$) and two different path-based centrality indices (closeness centrality $\mathcal {C}^\text{C}$ and betweenness centrality $\mathcal {C}^\text{B}$). According to strength centrality, a node is central if it is strongly connected to adjacent nodes. Eigenvector centrality considers the influence of a node on the network as a whole, where a node is considered central if the nodes connected to it are also central. A node with a high closeness centrality is central as information from this node can reach all other nodes in the network via short paths, and so the node can exert a more direct influence over the network. A node with a high betweenness centrality acts as a bridge between other parts of the network. $\mathcal {C}^\text{S}$ and $\mathcal {C}^\text{C}$ are more sensitive to local aspects of the network, as they only consider edges immediately connected to the investigated node. On the other hand, $\mathcal {C}^\text{E}$ and $\mathcal {C}^\text{B}$ are more sensitive to global aspects, as they consider all edges in the network when determining the centrality of any node.

Our long-term aim is to achieve a more general understanding of how the evolving epileptic brain network changes prior to seizures and how these changes relate to the emergence of seizures from subjects with epilepsy whose seizures originated from different brain regions (“Methods” section). To this end, and in order to avoid making any assumptions about a possible influence of the underlying structural and functional aspects of the respective pathologies, we here pooled the data of the heterogeneous group of subjects with pharmacoresistant epilepsies and used multiple centrality indices. We then investigated undirected, weighted evolving epileptic brain networks which we inferred from multi-day, multi-electrode intracranial electroencephalographic recordings (“Methods” section).

**Figure 1:**
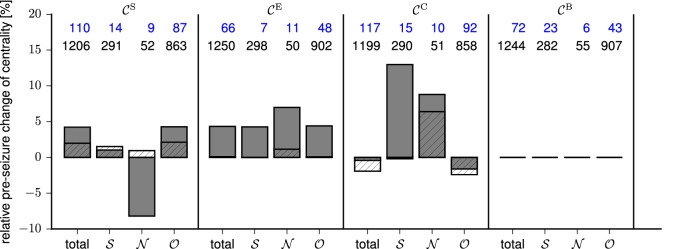
Relative pre-seizure change of centrality values of nodes in the different modules. Non-hatched/hatched bars represent median values over predictive/non-predictive nodes (median pre-seizure centrality values referenced against median centrality values from seizure-free periods). Blue/black numbers on top represent the number of predictive/non-predictive nodes in each module ($\mathcal {C}^\text{S}$ strength centrality; $\mathcal {C}^\text{B}$ betweenness centrality; $\mathcal {C}^\text{C}$ closeness centrality; $\mathcal {C}^\text{E}$ eigenvector centrality; “total” refers to the sum of these nodes). As betweenness centrality often yields values of 0, calculating a relative difference is not always possible, therefore we refer to the median absolute value which here amounts to 0.005 independent of the module (SOZ: $\mathcal {S}$, neighbours: $\mathcal {N}$, others: $\mathcal {O}$).

## Results

Given the individualised clinical evaluation, number and anatomical locations of intracranial electrodes were highly variable between subjects (“Methods” section). For this reason, we assigned electrode contacts to functional modules (seizure onset zone (SOZ) $\mathcal {S}$, direct neighborhood of SOZ $\mathcal {N}$, and all remaining contacts (others) $\mathcal {O}$; “Methods” section)^19^.

Borrowing statistical concepts from seizure prediction to identify nodes that carry predictive information of an impending seizure (“Methods” section), we find that different centrality indices (“Methods” section) generally identified different nodes as predictive, as expected. Out of 1316 total nodes, 227 (17%) were found to be predictive with at least one centrality (110 with $\mathcal {C}^\text{S}$, 66 with $\mathcal {C}^\text{E}$, 117 with $\mathcal {C}^\text{C}$ and 72 with $\mathcal {C}^\text{B}$). On the level of functional modules, each sampled brain region was frequently identified as predictive by multiple centralities, and functional module others $\mathcal {O}$ was identified most commonly even when correcting for the high variability of the electrode contacts in each functional module. This finding concurs with magnetic resonance imaging (MRI) studies in other subjects with epilepsy, which have revealed structural abnormalities outside of and even contralateral to the SOZ in multiple aetiologies of epilepsy^20–23^.

We investigated how the centrality of nodes changed during the pre-seizure period. To this end, we calculated the medians of the distributions of centrality values from the pre-seizure and the seizure-free period (for each node and centrality index respectively). We used the relative difference between the distributions’ median values to determine whether centrality values, on average, increased or decreased prior to seizures. As summarised in Fig. 1, we generally observed an increase of centrality values prior to seizures, except in the case of $\mathcal {C}^\text{S}$ for nodes in the functional module neighbours $\mathcal {N}$ and $\mathcal {C}^\text{C}$ for nodes in the functional module others $\mathcal {O}$. For nodes that were not predictive, we generally observed a less pronounced, but qualitatively comparable change than for predictive nodes (except for nodes in module $\mathcal {N}$ when using $\mathcal {C}^\text{S}$).

With the aforementioned predictive changes in centrality values, we next investigated whether the observed increase is associated with a re-ordering of node importance within the epileptic brain network. To determine the relative importance of predictive nodes, they were ranked by average centrality value (for each centrality separately, for seizure-free and pre-seizure periods respectively). Interestingly, predictive nodes were neither the most nor the least important ones but ranked among the top of the lower half. Moreover there was no significant difference between average rank of these nodes from pre-seizure and seizure-free periods for any centrality individually. This may indicate that pre-seizure changes are not necessarily confined to specific brain regions, but rather that there is, on average, an increase in interaction strength between all nodes in the epileptic brain network prior to seizures, consistent with findings in previous studies^14,19^.

**Figure 2:**
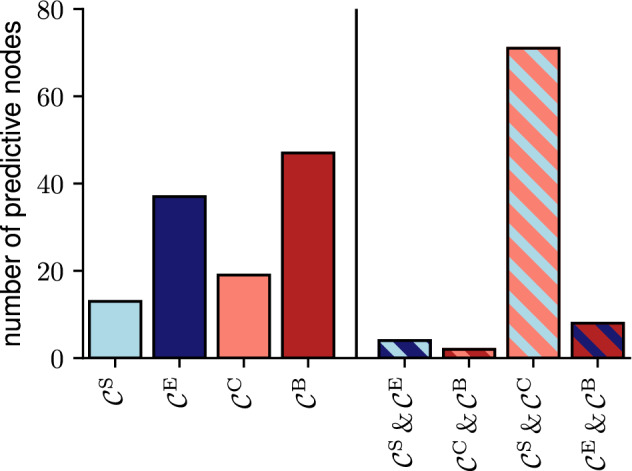
Predictive nodes as identified with only respective centralities or combinations of such. For example, there are 13 nodes identified as predictive with $\mathcal {C}^\text{S}$, that are not identified as predictive with the other three centralities, and 4 nodes identified as predictive with $\mathcal {C}^\text{S}$ and $\mathcal {C}^\text{E}$, that are not identified as predictive with the other two centralities. Different colours indicate different centralities (light blue: strength centrality $\mathcal {C}^\text{S}$; dark blue: eigenvector centrality $\mathcal {C}^\text{E}$; light red: closeness centrality $\mathcal {C}^\text{C}$; dark red: betweenness centrality $\mathcal {C}^\text{B}$). Centrality indices considering local/global aspects of the evolving epileptic brain network are depicted in light/dark colour respectively, while strength-/path-based centrality indices are depicted in blue/red. Hatched bars indicate a combination of the respective centralities (see colours above).

Subsequently, we investigated whether different centrality indices identify the same nodes as predictive. While we find that a majority of nodes are identified as predictive with only one centrality index, unexpectedly, a substantial number of nodes were identified as predictive with two or more indices (see Fig. 2). To further investigate the information gain from using multiple centrality indices, we separated the 227 predictive nodes into groups according to the centrality index or indices with which they were identified as predictive. Betweenness centrality and eigenvector centrality each identified the largest number of nodes as predictive (47 and 37 nodes, respectively) followed by closeness centrality (19 nodes) and strength centrality (13 nodes). It is to be noted that even two centrality indices based on the same network-theoretical concept (interaction-strength- or path-based), rarely identified the same predictive nodes. On the other hand, the largest group of nodes identified as predictive were congruently found with strength centrality and closeness centrality (a total of 71 nodes). Of note, this is a combination of two different network-theoretical concepts, which both consider local network characteristics. More rarely were nodes identified as predictive with combinations of three or all four centralities, which indicates that typically only some and not all aspects of the evolving epileptic brain network change during the pre-seizure period.

**Figure 3:**
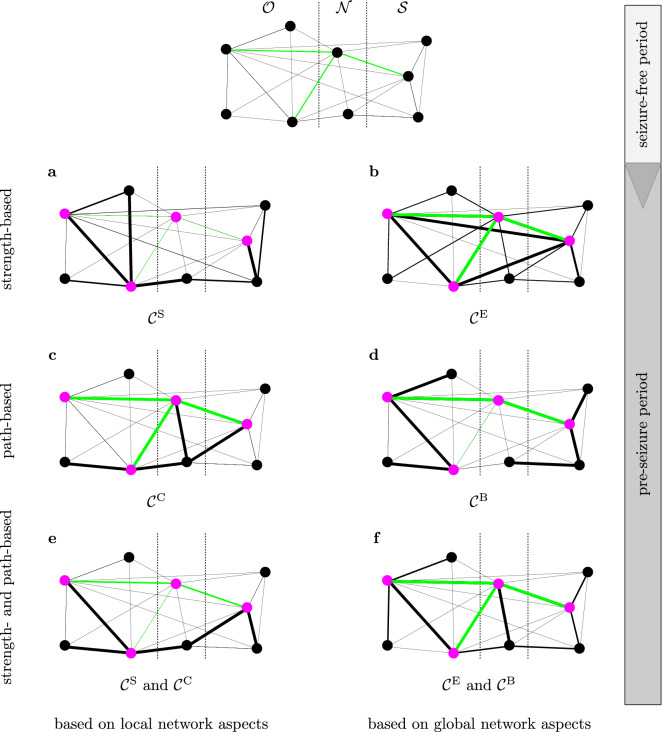
Scenarios for the pre-seizure reconfiguration of the evolving epileptic brain network. Schematic of the network divided into the three functional modules (others $\mathcal {O}$, neighbours $\mathcal {N}$, SOZ $\mathcal {S}$; separated by dashed lines). The different sub-figures (**a**–**f**) represent how the network during the seizure-free period (top) would change prior to seizures if the pink nodes were deemed predictive with the respective local and global interaction-strength-based and path-based centrality indices or combinations thereof (note that different centrality indices generally identified different nodes as predictive; we here restrict ourselves to just a few nodes to simplify visualisation). The networks can be assumed to be fully connected, however, for the purpose of visualisation, edges that remain unchanged during seizure-free and pre-seizure periods are not shown. Shortest paths identified in the seizure-free period (examples) are marked green. The thickness of an edge represents its edge weight: the thicker an edge the shorter the path traversing the edge or the stronger the connection between nodes. ($\mathcal {C}^\text{S}$: strength centrality; $\mathcal {C}^\text{E}$ eigenvector centrality; $\mathcal {C}^\text{C}$ closeness centrality; $\mathcal {C}^\text{B}$: betweenness centrality).

Given these findings, we propose several major scenarios for a pre-seizure reconfiguration of the evolving epileptic brain network, that can be inferred from significant differences between node centralities from the pre-seizure and seizure-free periods in the various functional modules (Fig. 3). In the following, we concentrate on the five most common occurrences of predictive nodes as identified with only respective centralities or combinations of such.

- **Scenario 1** (based on observations with strength centrality $\mathcal {C}^\text{S}$; Fig. 3a): As already described in a previous study^14^, during the pre-seizure period a small number of nodes both related to the seizure onset zone (SOZ) and brain regions far off the SOZ, become more strongly connected to the other nodes of the evolving epileptic brain network. Meanwhile, nodes related to the neighbourhood of the SOZ become less connected. Since we employed a synchronisation-based measure (mean phase coherence) to estimate the strength of interactions, this could indicate a loss of synchronisation, i.e., a decoupling of the neighbourhood from the rest of the network, while the latter interacts more strongly locally. A pre-seizure decrease in synchronisation has been hypothesised to be a state of increased susceptibility for pathological synchronisation during a seizure^24^ or depression of synaptic inhibition^25^, possibly allowing an easier transition to seizure activity. These findings could lead to the assumption that path structures traversing these nodes in the evolving epileptic brain network change prior to seizures. Surprisingly, however, as not all of these nodes carry predictive information (when assessed with closeness centrality $\mathcal {C}^\text{C}$ and betweenness centrality $\mathcal {C}^\text{B}$), path structures traversing these nodes remain unchanged. This indicates that the general exchange of information between brain regions remains largely unaffected during the pre-seizure period, which might explain the rare occurrence of epileptic prodromes^26,27^.
- **Scenario 2** (based on observations with eigenvector centrality $\mathcal {C}^\text{E}$; Fig. 3b): Beyond the local scope of strength centrality, our results obtained with the more global eigenvector centrality indicate that strongly connected nodes are strongly connected especially to each other prior to seizures, highlighting hub-like structures^28^. This is, however, not necessarily a formation of new hub-like structures, since their connection strength to the rest of the epileptic brain network does not change prior to seizures, as indicated by the lack of a significant change in their strength centrality. This is also supported by the fact that path structures traversing the hub-like structures remain unchanged. Moreover, since hub-like structures are not confined to any particular brain region (as also found in a structural study^29^), this might point to a recruitment of even brain regions assumed to be unaffected by the pathology, hereby contributing to the generation of seizure activity.
- **Scenario 3** (based on observations with closeness centrality $\mathcal {C}^\text{C}$; Fig. 3c): The shortening of the paths seen between nodes related to the SOZ and to its neighbourhood, as well as to the rest of the epileptic brain network prior to seizures, indicates that information can flow more easily along the paths connecting these network constituents. This is not necessarily accompanied by a profound increase in coupling between constituents (as indicated by $\mathcal {C}^\text{S}$) nor by a higher number of shortest paths traversing these nodes (as indicated by $\mathcal {C}^\text{B}$). However, as we can not infer the direction of this information flow with the methods applied here, several potential ictogenic mechanisms can be considered: e.g. nodes related to the SOZ recruit other nodes in the larger network into the generation of seizures^30,31^. This recruitment might also be facilitated by the fact that information flow between nodes far off the SOZ and its neighbourhood becomes less effective during the pre-seizure period (lengthening of the shortest paths). In contrast, nodes related to brain regions deemed unaffected by the pathology might recruit nodes related to the SOZ and/or its neighbourhood into the generation of seizures. Whatever the exact mechanism might be, these possibilities underline that the generation of seizures in any one part of the epileptic brain network is influenced by activity everywhere else in the network^4^.
- **Scenario 4** (based on observations with betweenness centrality $\mathcal {C}^\text{B}$; Fig. 3d): Expanding the previous interpretation^14^ of the formation of bottlenecks during the pre-seizure period, the unexpected lack of congruence between findings obtained with $\mathcal {C}^\text{C}$ and $\mathcal {C}^\text{B}$ (both centrality indices would identify the same node as relevant for the flow of information through the epileptic brain network) points to a general decrease in information flow (increased length of shortest paths), except through those nodes that become bottlenecks. On the one hand, this would indicate that the formation of bottlenecks can be regarded as an epiphenomenon, while on the other hand and since we here observed bottlenecks almost in the whole network these nodes possibly form a backbone of the evolving epileptic brain network.
- **Scenario 5** (based on observations with strength centrality $\mathcal {C}^\text{S}$ and closeness centrality $\mathcal {C}^\text{C}$; Fig. 3e): If we combine the information about pre-seizure changes in the epileptic brain network gained with local (and to a lesser extent also with global; Fig. 3f) path- and interaction-strength-based centrality indices, our findings point to groups of nodes associated with the SOZ and with brain regions far off the SOZ becoming more strongly connected prior to seizures, while the connection between these groups weakens and information flow within the whole network becomes hampered. This indicates a compartmentalisation of brain regions, which has been hypothesised to cause an increased vulnerability to the spreading of seizure activity^32^. Similar compartmentalisation has also been observed during seizures^33,34^. We speculate that the compartmentalisation seen before and during seizures results from the same underlying process.

## Conclusion

While previous studies already showed that the temporal change of node centrality—possibly induced by a reconfiguration of properties of evolving epileptic brain networks—can carry predictive information about impending seizures, we here aimed at a more comprehensive perspective of this reconfiguration. These networks—evolving, large-scale, fully connected networks (spanning lobes and hemispheres)—were constructed from iEEG data, with nodes representing the sampled brain regions and edges the time-varying functional interactions between them. By considering four different centrality indices (local and global interaction-strength-based and path-based indices), that reflect changes in the evolving epileptic brain network differently, and by using established statistical methods to identify nodes that carry predictive information^35^, we can now trace these changes which are specific to the pre-seizure period.

Pre-seizure changes in the network are not necessarily confined to specific brain regions, but rather there is, on average, a pre-seizure increase in interaction strength between all nodes in the epileptic brain network, consistent with findings in previous studies^14,19^. Moreover, with our proposed scenarios, we conclude that there is likely not a single network mechanism underlying ictogenesis. Rather, they point to local and global reconfigurations of the evolving large-scale epileptic brain network affecting virtually all network constituents, from the various brain regions to the (functional) connections between them.

An important limitation in this retrospective study was the high variability in implantation schemes for electrodes, which were purely clinically driven and relate to the structural and functional heterogeneity of the underlying disease. In many subjects with epilepsy, the area around the suspected SOZ is usually spatially oversampled, while data from other brain regions are often very limited or even absent. Even in subjects with greater electrode coverage, it is possible that evolving epileptic brain networks were incomplete as not all brain regions were sampled^15^. It is therefore possible that there are important regions for ictogenesis which lie outside of those considered in this study. Among others, there were several possible confounding influences on the distinction between dynamics from seizure-free and pre-seizure periods: subjects were often sleep-deprived and all had their individual antiepileptic medication dose tapered as part of the pre-surgical assessment. Furthermore, the possible impact of multi-day rhythms^36–38^ was not controlled for in this study, as data from multiple days were pooled for each subject.

Future studies should investigate the duration of the presumed pre-seizure period as a possible confounder. It is conceivable that there is a high inter-individual variation in pre-seizure period duration, which the variation in prodromal symptom onset and duration seems to support^39^. Finally, the results of this study should be combined with those of similar studies focusing on edges rather than nodes^19^, which could be expanded to include novel edge centrality indices^40^. Previous studies have assessed whether predictive edges connect predictive nodes^14^, reporting that this occurs in a majority of cases. A combination of information gained from predictive nodes and edges and their relation to the underlying anatomy and physiology could provide a more complete understanding of ictogenesis in evolving epileptic brain networks, could help to identify better targets for future treatment strategies^41–43^, and could support the translation of the network approach into clinical practice.

## Methods

### Data

In this retrospective study, we analysed multi-day, multi-electrode intracranial EEG (iEEG) recordings from 38 subjects with pharmacoresistant epilepsies with different anatomical origins (Table 1). The data were part of previous studies^8,14,19^. Between 2002 and 2012, 380 subjects with drug-resistant epilepsy underwent presurgical evaluation with intracranial electroencephalographic recordings. From this sample, we included subjects for which either a single or multiple seizure onset zones (SOZ) had been identified and resected, as well as subjects with multiple, non-resectable SOZs. We only included data from subjects if they had at least 18 h of recording that captured at least one seizure (with or without secondary generalisation). Recordings spanned an average of 83 h (total: 3239, range 18–228), and captured 2.5 clinical seizures on average (total: 99, range 1–7). Subclinical seizures were neglected in our analyses. Placement and number of electrodes were decided for each subject individually, and were entirely clinically driven (average number of contacts: $N=56$, range 16–120). The study was approved by the ethics committee of the University of Bonn, and all subjects with epilepsy had signed informed consent that their data could be used and published for research purposes. A parent or legal guardian gave written informed consent on behalf of the participant if below the age of 18. All experiments were performed in accordance with relevant guidelines and regulations.

For cortical surface recordings, subdural strip electrodes (four or eight platinum contacts with an intercontact distance of 10 mm) and/or subdural grid electrodes ($8 \times 4$ or $8 \times 8$ platinum contacts with an intercontact distance of 10 mm) were used. These types of electrodes were implanted in 74% of subjects. For recordings from the mesial temporal lobes, depth electrodes (equipped with 10 or 8 cylindrical contacts of nickel–chromium alloy; length: 2.5 mm, intercontact distance: 4 mm) were implanted using either a trans-occipital (10-contact electrodes) or orthogonal-to-the-mesial-structures approach (8-contact electrodes) to the hippocampus^44^. In five subjects, 8-contact depth electrodes were also implanted into lesions or focal cortical dysplasias. iEEG data were sampled at 200 Hz using a 16 bit analogue-to-digital converter, band-pass filtered between 0 and 45 Hz (4th order Butterworth characteristic), and a notch filter was used to suppress the power line frequency (50 Hz). Recorded signals were referenced against the average of two contacts which were selected for each subject individually, located distant from the suspected seizure onset zone (SOZ).

Seizures were identified by board-certified epileptologists on the iEEG and concomitant video recording. We divided data into pre-seizure and seizure-free periods. Recordings within the 4 h preceding an electrophysiologically defined seizure event were classified as pre-seizure^45^. Recordings within the 30 min after seizure onset were excluded from this analysis in order to not bias our analyses with effects from the seizure and particularly from the post-ictal period^46,47^. All remaining recording data were considered to be from the seizure-free period. Following pre-surgical analysis, board-certified epileptologists identified at least one SOZ in all subjects, being the region where electrical seizure activity was first identified. The electrode contacts within this SOZ were labelled as $\mathcal {S}$ for subsequent analyses. Electrode contacts not more than two contacts distant to the SOZ (“neighbours” or $\mathcal {N}$) were also considered separately to other electrode contacts more distant to the SOZ (“others” or $\mathcal {O}$). These classifications are subsequently referred to as “functional modules”^19^.

**Table 1:** Subject demographics. *Age* age at time of presurgical evaluation, *Dur* duration of epilepsy in years, *MRI* MRI findings (*w.p.f.* without pathological findings, *AHS* Ammon’s horn sclerosis, *bilat.* bilateral, *FCD* focal cortical dysplasia), *L* left, *R* right, *Loc* location of seizure onset zone (*MT* mesial temporal, *SMA* supplementary motor area, *P* parietal, *F* frontal, *Fpo* frontopolar, *Fpa* frontoparietal, *FT* frontotemporal, *T* temporal), *Out* epilepsy surgery outcome scale ^48^ (no surgery performed if empty entry), *Sz*r number of clinical seizures; $D_{tot}$ total recording duration in hours, $D_{int}$ total duration of seizure-free periods in hours, $D_{pre}$ total duration of pre-seizure periods in hours; *N* total number of electrode contacts, $N_{\mathcal {S}}$ number of electrode contacts in functional module “SOZ”; $N_{\mathcal {N}}$ number of electrode contacts in functional module “neighbours”, $N_{\mathcal {O}}$ number of electrode contacts in functional module “others”, $\#\mathcal {C}_\text {pred}$ number of centralities that identified predictive nodes.

Subj.	Age	Sex	Dur	MRI	Loc	Out	Szr	D$_\text {tot}$	D$_\text {int}$	D$_\text {pre}$	*N*	$N_{\mathcal {S}}$	$N_{\mathcal {N}}$	$N_{\mathcal {O}}$	$\#\mathcal {C}_\text {pred}$
1	54	Male	46	R AHS	RMT	2B	1	228	224	4	86	3	2	81	0
2	34	Male	29	L FCD	LF	1A	7	111	85	26	26	5	5	16	4
3	15	Female	10	R AHS	LT,RT		4	162	146	16	66	44	0	22	0
4	45	Female	42	L AHS	LT	1A	1	146	142	4	48	12	2	34	0
5	25	Female	21	w.p.f.	RMT	1A	1	82	78	4	58	10	1	47	0
6	22	Male	23	w.p.f.	RMT	1A	5	94	74	20	74	10	1	63	0
7	57	Male	51	Hamartia	RFPo	1A	3	71	61	10	72	14	11	47	3
8	39	Female	11	R AHS	RT	1A	3	91	79	12	52	11	3	38	0
9	24	Female	23	AHS bilat.	LMT,RMT		2	20	14	6	42	20	0	22	1
10	34	Male	33	L AHS, L FCD	LMT	1A	4	70	54	16	52	20	4	28	0
11	25	Male	24	L AHS	LMT	1A	3	26	17	9	58	3	5	50	0
12	43	Female	27	w.p.f.	LT	1A	3	94	85	9	56	8	0	48	4
13	29	Male	17	L AHS	LMT,RMT		4	92	76	16	120	20	4	96	1
14	38	Male	15	AHS bilat.	LMT	1A	2	52	44	8	46	8	4	34	0
15	44	Female	31	L FCD	LF	1A	1	103	99	4	14	4	0	10	0
16	52	Male	52	L AHS	LMT	1A	1	49	45	4	42	5	4	33	3
17	45	Male	24	w.p.f.	LT,RT		3	116	107	9	72	28	0	44	2
18	31	Female	14	w.p.f.	RT	1A	2	74	69	5	36	11	1	24	3
19	25	Female	6	w.p.f.	LMT,RMT		5	161	142	19	90	8	1	81	4
20	53	Female	13	L AHS	LP	1A	1	46	42	4	24	11	3	10	0
21	62	Female	50	Dysplasia	RFPa		3	94	84	10	56	39	1	16	2
22	44	Female	30	L AHS	LT,RT	1A	3	129	117	12	46	30	0	16	2
23	25	Male	13	R FCD	RFP	1A	3	18	8	10	30	5	4	21	2
24	26	Female	10	Dysplasia	LT	1A	1	26	22	4	16	5	4	7	1
25	54	Female	49	R FCD	RT	1A	1	67	63	4	62	9	7	46	0
26	27	Female	16	w.p.f.	LMT	1A	2	163	155	8	48	10	2	36	4
27	28	Female	25	R AHS	LMT,RMT		2	126	121	5	46	21	1	24	0
28	19	Male	9	AHS bilat.	LFT,RFT		2	47	40	7	78	34	2	42	2
29	26	Female	18	w.p.f.	LMT	2A	3	97	85	12	36	10	0	26	1
30	37	Male	5	R AHS	RMT	1A	2	103	95	8	46	10	4	32	4
31	25	Male	26	L AHS	LFT,RFT		2	32	25	7	78	0	0	78	0
32	37	Male	2	w.p.f.	LMT	1A	4	68	52	16	65	6	0	59	2
33	15	Female	11	L FCD	LFPo	1A	2	36	28	8	30	8	7	15	1
34	24	Male	4	w.p.f.	LMT	1A	2	67	59	8	65	6	3	56	4
35	22	Male	18	Lesion	LFT	1A	3	19	7	12	38	4	2	32	2
36	29	Female	12	w.p.f.	LMT,RMT		2	37	29	8	88	6	1	81	3
37	41	Female	13	w.p.f.	LMT,RM		2	127	119	8	118	13	5	100	4
38	27	Female	13	L FCD	LSMA	1A	2	67	59	8	30	6	7	17	0

Subjects received different antiepileptic drugs (AEDs) with different mechanisms of action, and the majority of subjects were under combination therapy with two or more AEDs. During presurgical evaluation AEDs were reduced in a subject-specific manner, and many subjects did not have discontinuation of all AEDs.

### Identifying evolving epileptic brain networks

Following previous studies (e.g.^14,19,45^), we used a sliding window approach and estimated the strength of time-varying functional interactions between brain regions *n* and *m* $((n,m) = 1,\ldots , N)$ sampled by the implanted electrodes, using mean phase coherence^49^:

$$\begin{aligned} R_{nm}=\left| \frac{1}{T}\sum _{j=0}^{T-1}{e^{{i}\left( \Phi _n(j)-\Phi _m(j)\right) }}\right| . \end{aligned}$$

*T* is the number of data points per window and $\Phi _n$ is the instantaneous phase time series of node *n* that we derived from the Hilbert transform of the iEEG time series of node *n*. An important property of this analytic signal approach (particularly in case of two or more superimposed oscillatory components) is that the instantaneous frequency relates to the predominant frequency in the Fourier spectrum^50,51^. Since the predominant frequency may be subject to fluctuations in the iEEG time series, the instantaneous frequency can vary rhythmically around the predominant frequency resulting in spurious estimates of the instantaneous phase. Such effects can nevertheless be reduced, e.g., by taking the temporal average. Note that from an electrophysiological point of view, it might be more reasonable to look adaptively (e.g., via the Hilbert transform) at interactions between predominant rhythms in the iEEG than to look at interactions in some a priori fixed frequency bands (e.g., via wavelet) for which there is no power in the time series^51,52^. $R_{nm}$ falls within the range [0, 1], where $R_{nm} = 1$ indicates fully phase-synchronised brain regions, while $R_{nm} = 0$ indicates no phase synchronisation.

A non-overlapping sliding-window with $T = 4096$ data points (20.48 s duration) was used to calculate $R_{nm}$ for all possible combinations of brain regions (nodes (*n*, *m*)). Mean phase coherence values were used as edge weights in subsequent network analysis, while electrode contacts represented nodes, resulting in a sequence of undirected, weighted and fully connected epileptic brain networks.

### Estimating node centrality indices

For each node in the evolving epileptic brain network , we calculated four different centralities: strength centrality ($\mathcal {C}^\text{S}$; which is equivalent to degree centrality in unweighted networks^53,54^), eigenvector centrality ($\mathcal {C}^\text{E}$), closeness centrality ($\mathcal {C}^\text{C}$), and betweenness centrality ($\mathcal {C}^\text{B}$). This calculation was repeated for each time-window, in order to assess changes in a node’s centrality over time.

According to strength centrality, a node is central if it is strongly connected to adjacent nodes, and is defined as the summed weights of edges connected to the node:

$$\begin{aligned} \mathcal {C}^\text{S}(n)=\sum _{m=1}^{N} R_{nm}, \end{aligned}$$

where $R_{nm}$ is the weight of the edges connecting nodes *n* and *m*, and nodes *n* and *m* are adjacent.

Eigenvector centrality considers the influence of a node on the network as a whole, where a node is considered central if the nodes connected to it are also central, and is defined as

$$\begin{aligned} \mathcal {C}^\text{E}(n)=\frac{1}{\lambda _{\max }}\sum _{m=1}^{N} R_{nm}\,\mathcal {C}^\text{E}(m), \end{aligned}$$

where $\lambda _{\max }$ is the dominant eigenvalue of the weighted adjacency matrix, $R_{nm}$ is the weight of edges between nodes *n* and *m*, and $\mathcal {C}^\text{E}(m)$ is the eigenvector centrality of node *m*. This equation is applied iteratively until eigenvector centrality values remain stable.

Closeness centrality considers the distance between a node and all other nodes in the network. A node with a high closeness centrality is central as information from this node can reach all other nodes in the network via short paths, and so the node can exert a more direct influence over the network. $\mathcal {C}^\text{C}$ is calculated as follows:

$$\begin{aligned} \mathcal {C}^\text{C}(n)=\frac{1}{\sum _{m=1}^{N}d_{nm}}, \end{aligned}$$

where $d_{nm}$ is the length of the shortest path between nodes *n* and *m*, calculated as the sum of the inverse of all edge weights on the path.

Finally, betweenness centrality is a measure of how frequently a given node falls on the shortest path between two other nodes. A node with a high betweenness centrality is central because it acts as a bridge between other brain regions. Betweenness centrality of a node *n* is given by

$$\begin{aligned} \mathcal {C}^\text{B}(n)=\frac{2}{(N-1)(N-2)}\sum _{l\ne m\ne n}\frac{q_{lm}(n)}{G_{lm}}, \end{aligned}$$

where $G_{lm}$ is the number of shortest paths between nodes *l* and *m*, and $q_{lm}(n)$ is the number of shortest paths between nodes *l* and *m* which pass through node *n*. The length of a path is calculated as the sum of the inverse of all edge weights on that path.

Both $\mathcal {C}^\text{C}$ and $\mathcal {C}^\text{B}$ consider shortest paths in some sense. A path between two nodes describes a series of edges (which can be just one edge) that are traversed when going from one node to the other. A path is considered short or strong (long or weak) if the sum of the inverse edge weights along this path is small (large). Accordingly, we employed two different interaction-strength-based centrality indices ($\mathcal {C}^\text{S}$ and $\mathcal {C}^\text{E}$) and two different path-based centrality indices ($\mathcal {C}^\text{C}$ and $\mathcal {C}^\text{B}$). While eigenvector centrality was iteratively calculated for all nodes in the network, and thus takes into account more global aspects of the network, strength centrality only considers the strength of interactions of a given node to its adjacent ones, reflecting only local aspects of the network. In case of betweenness centrality, the global path structure in the network is considered (by identifying all shortest paths) when estimating the centrality of a node, while for closeness centrality only local path structures are considered, namely the shortest paths from the node, for which the centrality is estimated, to every other node in the network. Hence, $\mathcal {C}^\text{S}$ and $\mathcal {C}^\text{C}$ are more sensitive to local aspects of the network compared to $\mathcal {C}^\text{E}$ and $\mathcal {C}^\text{B}$. Note that the term local does not refer to a spatial relationship, as we estimated the centralities for fully connected networks, but to certain edges that are either directly connected to the node for which the centrality is calculated, or are a part of a shortest path connected to this node. $\mathcal {C}^\text{E}$ and $\mathcal {C}^\text{B}$ are sensitive to global aspects, as they consider all edges in the network, when determining the centrality of any node.

### A statistical approach to identify predictive nodes

In order to determine whether a node’s centrality changed prior to a seizure, we compared its distributions of values from pre-seizure and seizure-free periods using the Kolmogorov–Smirnov (KS) test. The *p*-values of this test were corrected for multiple comparisons (number of nodes) using the Bonferroni method. In order to be considered for further analysis, a node’s centrality had to significantly differ between pre-seizure and seizure-free periods ($p < 0.05$).

In order to verify the specificity of this change, and to minimise the impact of confounding variables such as the influence of rhythmic fluctuations in interaction strength^8^, seizure time surrogates (STS) were created to compare the real data to^55^. 19 STS time-lines were created for each subject, where “seizure times” were placed randomly within the seizure-free periods, but maintained the same total number of seizures and the distribution of intervals between sequential seizures. The KS test was then repeated for each of these STS datasets. If the test revealed larger KS-statistic values (the largest distance between two cumulative distributions) when comparing centrality values of pre-seizure to seizure-free periods for the STS than for the real data, then any difference found in the real data could be explainable by changes of node centrality due to unrelated fluctuations in network topology, e.g. measurement errors or daily rhythms. Using this method we determined the number of predictive nodes. In order to be identified as predictive, a node’s KS-statistic value had to be at least 5% greater (to compensate for estimation errors) than any of its KS-statistic values for STS for at least one centrality (note that the KS-statistic is not sensitive to the direction of change). Given the different sizes (number of electrode contacts, Table 1) of functional modules within subjects, the hypergeometric statistic was used to test whether more nodes located within one module were predictive than expected by chance ($p < 0.05$).

With any of the employed centrality indices, we identified at least one predictive node in 23 of 38 subjects with epilepsy (Table 1), and only data from these subjects will be considered in subsequent analyses. Statistical analysis found no significant correlation between the identification of predictive nodes and the subjects’ age, sex, duration of epilepsy, surgery outcome, location of the SOZ (hemisphere and lobe), or number of electrodes. We note that our findings are neither dominated by data from a single nor from few specific subjects.

## Data Availability

The data that support the findings of this study are available from the corresponding author upon reasonable request. The data are not publicly available as they contain information that could compromise the privacy of research participants.

## References

[1] World Health Organization (2019). Epilepsy: A Public Health Imperative.

[2] Kwan P, Schachter SC, Brodie MJ (2011). Drug-resistant epilepsy. N. Engl. J. Med..

[3] de Tisi J (2011). The long-term outcome of adult epilepsy surgery, patterns of seizure remission, and relapse: a cohort study. Lancet.

[4] Spencer SS (2002). Neural networks in human epilepsy: evidence of and implications for treatment. Epilepsia.

[5] Berg AT, Scheffer IE (2011). New concepts in classification of the epilepsies: entering the $$21^{\rm st} $$ century. Epilepsia.

[6] Richardson MP (2012). Large scale brain models of epilepsy: dynamics meets connectomics. J. Neurol. Neurosurg. Psychiatry.

[7] Bernhardt BC, Bonilha L, Gross DW (2015). Network analysis for a network disorder: the emerging role of graph theory in the study of epilepsy. Epilepsy Behav..

[8] Dickten H, Porz S, Elger CE, Lehnertz K (2016). Weighted and directed interactions in evolving large-scale epileptic brain networks. Sci. Rep..

[9] Geier C, Bialonski S, Elger CE, Lehnertz K (2015). How important is the seizure onset zone for seizure dynamics?. Seizure.

[10] Geier C, Lehnertz K (2017). Long-term variability of importance of brain regions in evolving epileptic brain networks. Chaos.

[11] Wilke C, Worrell G, He B (2011). Graph analysis of epileptogenic networks in human partial epilepsy. Epilepsia.

[12] van Mierlo P (2013). Ictal-onset localization through connectivity analysis of intracranial EEG signals in patients with refractory epilepsy. Epilepsia.

[13] Burns SP (2014). Network dynamics of the brain and influence of the epileptic seizure onset zone. Proc. Natl. Acad. Sci. U.S.A..

[14] Rings T, von Wrede R, Lehnertz K (2019). Precursors of seizures due to specific spatial-temporal modifications of evolving large-scale epileptic brain networks. Sci. Rep..

[15] Kuhlmann L, Lehnertz K, Richardson MP, Schelter B, Zaveri HP (2018). Seizure prediction—ready for a new era. Nat. Rev. Neurol..

[16] Borgatti SP, Everett MG (2006). A graph-theoretic perspective on centrality. Soc. Netw..

[17] Lü L (2016). Vital nodes identification in complex networks. Phys. Rep..

[18] Newman M (2018). Networks.

[19] Lehnertz K, Dickten H, Porz S, Helmstaedter C, Elger CE (2016). Predictability of uncontrollable multifocal seizures—towards new treatment options. Sci. Rep..

[20] Yu O, Mauss Y, Namer I, Chambron J (2001). Existence of contralateral abnormalities revealed by texture analysis in unilateral intractable hippocampal epilepsy. Magn. Res. Imaging.

[21] Seidenberg M (2005). Ipsilateral and contralateral MRI volumetric abnormalities in chronic unilateral temporal lobe epilepsy and their clinical correlates. Epilepsia.

[22] Lin JJ (2007). Reduced neocortical thickness and complexity mapped in mesial temporal lobe epilepsy with hippocampal sclerosis. Cereb. Cortex.

[23] Hallbook T (2010). Contralateral MRI abnormalities in candidates for hemispherectomy for refractory epilepsy. Epilepsia.

[24] Mormann F (2003). Epileptic seizures are preceded by a decrease in synchronization. Epilepsy Res..

[25] Quyen MLV (2005). Preictal state identification by synchronization changes in long-term intracranial EEG recordings. Clin. Neurophysiol..

[26] Mormann F, Lehnertz K, Reuber M, Schachter SC (2013). Epileptic prodromes. Borderland of Epilepsy Revisited.

[27] Besag FM, Vasey MJ (2018). Prodrome in epilepsy. Epilepsy Behav..

[28] Khambhati AN (2015). Dynamic network drivers of seizure generation, propagation and termination in human neocortical epilepsy. PLoS Comput. Biol..

[29] Yasuda CL (2015). Aberrant topological patterns of brain structural network in temporal lobe epilepsy. Epilepsia.

[30] Spencer DD, Gerrard JL, Zaveri HP (2018). The roles of surgery and technology in understanding focal epilepsy and its comorbidities. Lancet Neurol..

[31] Zaveri HP (2020). Controversies on the network theory of epilepsy: debates held during the ICTALS 2019 conference. Seizure.

[32] Netoff TI, Clewley R, Arno S, Keck T, White JA (2004). Epilepsy in small-world networks. J. Neurosci..

[33] Kramer MA, Kolaczyk ED, Kirsch HE (2008). Emergent network topology at seizure onset in humans. Epilepsy Res..

[34] Schindler K, Bialonski S, Horstmann M-T, Elger CE, Lehnertz K (2008). Evolving functional network properties and synchronizability during human epileptic seizures. Chaos.

[35] Mormann F, Andrzejak R, Elger CE, Lehnertz K (2007). Seizure prediction: the long and winding road. Brain.

[36] Karoly PJ (2017). The circadian profile of epilepsy improves seizure forecasting. Brain.

[37] Baud MO (2018). Multi-day rhythms modulate seizure risk in epilepsy. Nat. Commun..

[38] Karoly PJ (2018). Circadian and circaseptan rhythms in human epilepsy: a retrospective cohort study. Lancet Neurol..

[39] Scaramelli A (2009). Prodromal symptoms in epileptic patients: clinical characterization of the pre-ictal phase. Seizure.

[40] Bröhl T, Lehnertz K (2019). Centrality-based identification of important edges in complex networks. Chaos.

[41] Englot DJ, Birk H, Chang EF (2017). Seizure outcomes in nonresective epilepsy surgery: an update. Neurosurg. Rev..

[42] Schulze-Bonhage A (2017). Brain stimulation as a neuromodulatory epilepsy therapy. Seizure.

[43] Nagai Y (2018). Epileptic seizures are reduced by autonomic biofeedback therapy through enhancement of fronto-limbic connectivity: A controlled trial and neuroimaging study. EBioMedicine.

[44] Sanz-Garcia A, Rings T, Lehnertz K (2018). Impact of type of intracranial EEG sensors on link strengths of evolving functional brain networks. Physiol. Meas..

[45] Mormann F (2005). On the predictability of epileptic seizures. Clin. Neurophysiol..

[46] Helmstaedter C, Elger CE, Lendt M (1994). Postictal courses of cognitive deficits in focal epilepsies. Epilepsia.

[47] So NK, Blume WT (2010). The postictal EEG. Epilepsy Behav..

[48] Engel J, van Ness PC, Rasmussen TB, Ojemann LM, Engel J (1993). Outcome with respect to epileptic seizures. Surgical Treatment of the Epilepsies.

[49] Mormann F, Lehnertz K, David P, Elger CE (2000). Mean phase coherence as a measure for phase synchronization and its application to the EEG of epilepsy patients. Physica D.

[50] Boashash B (1992). Time Frequency Signal Analysis: Methods and Applications.

[51] Frei MG (2010). Controversies in epilepsy: debates held during the fourth international workshop on seizure prediction. Epilepsy Behav..

[52] Osterhage H, Mormann F, Staniek M, Lehnertz K (2007). Measuring synchronization in the epileptic brain: a comparison of different approaches. Int. J. Bifurcat. Chaos Appl. Sci. Eng..

[53] Barrat A, Barthélemy M, Pastor-Satorras R, Vespignani A (2004). The architecture of complex weighted networks. Proc. Natl. Acad. Sci. U.S.A..

[54] Kuhnert M-T, Geier C, Elger CE, Lehnertz K (2012). Identifying important nodes in weighted functional brain networks: a comparison of different centrality approaches. Chaos.

[55] Andrzejak RG (2003). Testing the null hypothesis of the nonexistence of a preseizure state. Phys. Rev. E.

